# An evaluation of ankle–brachial blood pressure index in adult Nigerians with sickle cell anaemia

**DOI:** 10.5830/CVJA-2011-013

**Published:** 2012-02

**Authors:** NI Oguanobi, BJC Onwubere, SO Ike, EC Ejim, OG Ibegbulam, O Agwu

**Affiliations:** Department of Medicine, University of Nigeria Teaching Hospital, Enugu, Nigeria; Department of Medicine, University of Nigeria Teaching Hospital, Enugu, Nigeria; Department of Medicine, University of Nigeria Teaching Hospital, Enugu, Nigeria; Department of Medicine, University of Nigeria Teaching Hospital, Enugu, Nigeria; Department of Haematology, University of Nigeria Teaching Hospital, Enugu, Nigeria; Department of Haematology, University of Nigeria Teaching Hospital, Enugu, Nigeria

**Keywords:** ankle–brachial blood pressure index, adult Nigerians, sickle cell anaemia

## Abstract

**Aim:**

There are few studies to be found in the literature on ankle–brachial index in sickle cell disease. The aim of this study was to compare ankle–brachial index of steady-state adult sickle cell anaemia patients with that of normal controls.

**Methods:**

A descriptive cross-sectional study of 62 sickle cell anaemia patients and 62 age- and gender-matched normal controls was carried out in the adult outpatient sickle cell clinics and the cardiac centre of the University of Nigeria Teaching Hospital (UNTH), Enugu, Nigeria from February to August 2007. The supine brachial and ankle blood pressures were measured separately with the cuff of the mercury sphygmomanometer applied to the right arm and right calf, respectively.

**Results:**

The ankle systolic blood pressure was lower in patients with sickle cell anaemia than in the controls (*p* < 0.001). The mean indices for ankle–brachial index were 0.88 ± 0.09 and 1.03 ± 0.06, respectively for patients and controls. This difference was statistically significant (*p* < 0.001). Seventy three per cent of the patients had ankle–brachial index less than 0.9 compared with controls (5%). This was also significant (*p* < 0.001).

## Abstract

Ankle–brachial blood pressure index is defined as the ratio of the brachial systolic blood pressure and the ankle systolic blood pressure. A lower ankle–brachial index (< 0.09) was associated with a 4.2-fold increase in relative risk of cardiovascular mortality in a study on asymptomatic male subjects when compared to those with a normal ankle–brachial index.^1^ The Strong Heart study showed that the adjusted hazard ratio for cardiovascular mortality in the ankle–brachial index (> 1.4) was 2.09 compared to those with a normal ankle–brachial index.^2^

An ankle–brachial blood pressure index greater than 1.3 indicates poor compressibility of the arteries and signifies the presence of arterial calcification, which is common in diabetes mellitus. This makes the diagnosis of peripheral vascular disease less reliable and is the main limitation of the ankle–brachial blood pressure index. The other limitation of note is where patients with a high-grade aorto–iliac stenosis or occlusion present with a normal ankle–brachial blood pressure index at rest due to the presence of a rich collateral network.^3^

When compared to the gold standard of angiography, hospital studies have shown an ankle–brachial blood pressure index less than 0.9 to be 95% sensitive in detecting healthy subjects.^4^ The diagnostic criteria for peripheral artery disease based on ankle–brachial blood pressure index described values of 0.9–1.3 as normal, 0.7–0.9 as indicating mild disease, 0.41–0.69 as moderate disease and ≤ 0.4 as severe disease.^5^

A Chinese population-based comparison of ankle–brachial blood pressure index determined from consecutive auscultatory or Doppler measurements at the posterior tibia and brachial arteries showed that the mean ankle–brachial blood pressure index values were significantly higher on Doppler than on auscultatory measurements, with intermediate levels on oscillometric determination. The differences between the three measurements were not homogenously distributed across the range of ankle–brachial blood pressure index values.^6^

Few studies on this subject are to be found in the literature. This study was aimed at comparing the ankle–brachial index of steady-state adult sickle cell anaemia patients with that of normal controls.

## Methods

A descriptive cross-sectional study of 62 sickle cell anaemia patients and 62 age- and gender-matched normal controls was carried out in the adult outpatient sickle cell clinics and the cardiac centre of the University of Nigeria Teaching Hospital (UNTH), Enugu, Nigeria from February to August 2007. The study subjects were drawn from adult patients (age ≥ 18 years)^7^ attending the adult sickle cell clinics of the UNTH, Enugu, who had haemoglobin genotype SS on haemoglobin electrophoresis, were in steady state and had consented to participate in the study. Steady state is defined as absence of any crisis in the preceding four weeks, and absence of any symptoms or signs attributable to acute illness.

The weight and height of each subject were recorded and the surface area was determined from a standard formula.^8^ The supine brachial and ankle blood pressures were measured separately with the cuff of the mercury sphygmomanometer applied to the right arm and right calf, respectively. The approximate systolic blood pressures were obtained by palpation of the brachial and the dorsalis pedis pulses. The cuff was deflated and re-inflated to about 10 mmHg above the approximate systolic value. Phase I and IV Korotkoff ’s sounds were used as systolic and diastolic blood pressure readings, respectively. Haematological parameters such as haematocrit, reticulocyte count, white blood cell count with differentials, and haemoglobin electrophoresis were obtained.

Data were presented as means ± standard deviation. In order to examine the effect of anaemia on the variables, the subjects were classified, based on the haematocrit values, into four classes in accordance with the World Health Organisation classification of anaemia, as follows: class 1, normal (haematocrit ≥ 36%); class 2, mild anaemia (haematocrit 30–35.9%); class 3, moderate anaemia (haematocrit 21–29.9%); class 4, severe anaemia (haematocrit 18–20.9%).^9^

Inter-class differences in blood pressures in the patients were compared by one-way analysis of variance and *post hoc* multiple comparison of means using the Tukey’s honestly significant difference test. Intra-class differences in parameters between patients and controls in the same haematocrit class were analysed using the independent Student’s *t*-test. All statistical analyses were carried out using the Statistical Packages for Social Sciences (SPSS Inc, Chicago, Illinois) software version 11.0. Statistical tests with probability values less than 0.05 were considered statistically significant.

## Results

The age and gender distribution of the subjects are shown in Table 1. The mean ages for patients and controls were 28.27 ± 5.58 (range 18–44) and 28.37 ± 5.91 (range 18–45) years, respectively. There were no statistically significant age and gender differences in patients and controls. The study subjects had statistically significantly lower mean values than the controls in the measurement of height, weight, body mass index and body surface area (*p* < 0.001) (Table 1).

**Table 1:** Age, Gender And Anthropometric Data

*Parameters*	*SCA Mean (SD)*	*Controls Mean (SD)*	t-*test*	p-*value*
Age (years)	28.27 (5.58)	28.37 (5.91)	0.987	0.924
Gender [frequency (%)]				
Male	31 (50)	31 (50)	0.00	1.00^a^
Female	31 (50)	31 (50)
Total	62	62
Weight (kg)	54.97 (10.61)	67.35 (8.37)	7.20	< 0.001*
Height (m)	1.62 (0.14)	1.72 (0.07)	4.960	< 0.001*
Body surface area (m^2^)	1.62 (0.03)	1.78 (0.14)	3.723	< 0.001*
Body mass index (kg/m^2^)	20.47 (2.73)	23.87 (3.22)	6.181	< 0.001*

*Statistically significant, ^a^Chi-square. SCA: sickle cell anaemia.

The ankle systolic blood pressure was lower in sickle cell anaemia patients than in the controls (*p* < 0.001) (Table 2). The ratio of the systolic ankle-to-brachial blood pressure (ankle–brachial systolic blood pressure index) was compared in patients and controls (Table 3). The observed mean indices were 0.88 ± 0.09 and 1.03 ± 0.06, respectively for patients and controls. This difference was statistically significant (*p* < 0.001). Seventy three per cent (73.33%) of the patients had ankle–brachial index less than 0.9, compared with controls (5%). This was also significant (*p* < 0.001).

**Table 2:** Physiological Parameters In Sickle Cell Anaemia; Comparison With Haematocrit Levels

*Mean (SD) haematocrit levels*					
*Parameters*	*Mild*	*Moderate*	*Severe*	*F statistic*	p-*value*
Age	26.11 (3.59)	23.60 (5.35)	25.18 (7.48)	0.922	0.402
Pulse rate	90.89 (13.57)	86.45 (8.36)	89.54 (5.54)	1.214	0.304
Brachial SBP	115.11 (13.57)	122.85 (9.34)	110.91 (13.75)	6.029	0.004*
Brachial DBP	69.78 (12.21)	64.60 (8.84)	61.87 (4.05)	2.083	0.134
Pulse pressure	45.33 (10.28)	58.25 (12.19)	49.09 (12.21)	5.747	0.005*
MAP	74.53 (28.58)	83.42 (6.82)	78.49 (6.39)	2.210	0.119
Ankle SBP	116.67 (13.23)	112.90 (13.09)	96.54 (10.77)	8.373	0.001*
Ankle DBP	73.33 (12.25)	67.15 (10.17)	60.00 (6.32)	4.543	0.015*
Ankle/brachial index	1.01 (0.06)	0.90 (0.04)	0.87 (0.09)	3.260	0.046*

*Statistically significant, DBP: diastolic blood pressure, SBP: systolic blood pressure, MAP: mean arterial blood pressure.

**Table 3:** Physiologic Data In Patients And Controls

*Parameters*	*SCA Mean (SD)*	*Controls Mean (SD)*	t-*test*	p-*value*
Pulse rate (beat/min)	87.68 (8.91)	72.13 (6.79)	11.062	< 0.001*
Brachial systolic BP (mmHg)	119.50 (11.70)	121.2 (8.97)	0.527	0.599
Brachial diastolic BP (mmHg)	64.867 (8.95)	76.88 (6.18)	8.629	< 0.001*
Brachial pulse pressure (mmHg)	54.63 (12.87)	44.31 (10.91)	4.735	0.001*
Ankle BP (mmHg)	105.47 (14.23)*a* 66.77 (10.52)*b*	124.87 (9.32)*a* 71.67 (13.20)*b*	6.826 2.454	< 0.001* 0.016*
Ankle brachial systolic BP index	0.88 (0.09)	1.03 (0.06)	6.567	< 0.001*
Mean brachial arterial BP (mmHg)	81.18 (12.65)	91.71 (5.47)	5.850	< 0.001*
Haematocrit (%)	24.07 (3.10)	38.65 (1.97)	30.589	< 0.001*

*Statistically significant, ^a^systolic, ^b^diastolic. BP: blood pressure, SCA: sickle cell anaemia.

Table 2 compares the mean values for the physiological parameters in the patients in the three separate categories, based on their haematocrit levels, using analysis of variance. Significant differences in mean values were observed in the brachial systolic blood pressure (*F* = 6.029; *p* = 0.004), ankle systolic blood pressure (*F* = 8.373; *p* = 0.001), ankle diastolic blood pressure (*F* = 4.543; *p* = 0.015) and ankle–brachial index (*F* = 3.260; *p* = 0.046), as well as pulse pressure (*F* = 5.747; *p* = 0.005).

Pair-wise *post hoc* multiple comparisons of means using the Tukey’s honestly significant difference test showed that the observed difference in systolic blood pressure was accounted for by the difference in haematocrit between the moderate and severe anaemia groups (SE = 3.7128; *p* = 0.006). For the ankle systolic blood pressure, differences were significant in the mild versus severe anaemia group (SE = 5.7227; *p* = 0.002) and in the moderate versus severe anaemia group (SE = 4.3347; *p* = 0.001). The mean pulse pressure for patients with mild anaemia was significantly different from patients with moderate anaemia (SE = 4.40621; *p* = 0.0013).

## Discussion

The finding in this study of a significant reduction in ankle–brachial index in sickle cell anaemia is intriguing. Ankle–brachial index was found to be significantly lower in patients with severe anaemia (haematocrit 18–20.9%) than in patients with mild anaemia (haematocrit 30–35.9%). A comparison of ankle–brachial index in patients and controls with comparable haematocrit (30–35.9%) demonstrated no difference in values. This suggests the significant role of chronic anaemia in the reduction of ankle–brachial index in sickle cell anaemia, although the mechanism of this effect is difficult to explain. It is also possible that the subset of patients with severe anaemia may have represented patients with more severe disease and therefore more severe cardiovascular complications. Ankle–brachial index in this study did not significantly correlate with the age of the patients (duration of chronic anaemia) (Table 4) or with the frequency of crisis.

**Table 4:** Effect Of Age On Ankle–Brachial Index In Patients And Controls

*Ankle–brachial index*				
*Age range (years)*	*Patients*	*Controls*	t-*test*	p-*value*
18–22	0.945 (0.014)	1.041 (0.042)	2.081	0.052
23–27	0.933 (0.113)	1.041 (0.087)	3.859	0.001*
28–32	0.916 (0.116)	1.039 (0.037)	4.820	0.000*
33–44	0.859 (0.096)	1.014 (0.109)	3.088	0.015*

*Statistically significant.

Autopsy studies on sickle cell anaemia patients have reported fibromuscular dysplastic narrowing involving multiple small arteries, as well as intravascular plugs of sickled erythrocytes.^10^ Focal fibromuscular dysplasia has been found at many different sites in a variety of organs in non-sickling individuals.^11^-^13^ Whether fibromuscular dysplastic narrowing of arteries may contribute to reduction in ankle–brachial index in sickle cell anaemia remains to be studied.

A reduced ankle–brachial blood pressure index has been associated with significantly increased risk of cardiovascular disease and stroke that is independent of other risk factors.^1^,^2^ An earlier study had documented reduced ankle–brachial index in sickle cell patients who presented with leg ulcers.^14^

## Conclusion

A low ankle-to-brachial blood pressure ratio (< 0.9) could be a relatively easy-to-obtain marker of increased cardiovascular risk in patients with sickle cell anaemia. Further studies are recommended to evaluate the prognostic implications of reduced ankle–brachial index in patients with sickle cell anaemia.

## References

[1] Resnick HE, Lindsay RS, Mc Dermott MM (2007). Relationship of high and low ankle brachial index to all – cause and cardiovascular disease mortality. The Strong Heart Study.. Circulation.

[2] Konitzer M, Dramaix M, Sobolski J, Degre S, De Backer G (1995). Ankle/arm pressure index in asymptomatic middle-aged males; an independent predictor of ten-year coronary heart disease mortality.. Angiology.

[3] Belch JJ, Topol EJ, Agnelli G (2003). Prevention of atherothrombotic disease network, critical issues in peripheral arterial disease detection and management: a call to action.. Arch Intern Med.

[4] Bernstein EF, Fronek A (1983). Current status of non-invasive tests in the diagnosis of peripheral arterial disease.. Surg Clin North Am.

[5] Bhasin N, Scot DJA (2007). Ankle brachial pressure index: identifying cardiovascular risk and improving diagnostic accuracy.. J R Soc Med.

[6] Pan CR, Staessen JA, Li Y, Wang JG (2007). Comparison of three measures of the ankle–brachial blood pressure index in a general population.. Hypertens Res.

[R07] (1989). United Nations Convention on the right of the child. General Assembly resolution 44/24..

[8] Ganong WF (1989). Review of Medical Physiology..

[9] DeMaeyer EM (1989). Preventing and controlling iron deficiency anaemia through primary health care..

[10] James TN, Riddick L, Massing GK (1994). Sickle cells and sudden death; morphologic abnormalities of the cardiac conducting system.. J Lab Clin Med.

[R11] Hunt JC, Harrision EG, Kincaid OW, Bernatz PE, Daris GD (1962). Idiopathic fibrous and fibromuscular stenoses of the renal arteries associated with hypertension..

[12] Hartman JD, Young 1, Bank AA, Rosenblatt SA (1971). Fibromuscular hyperplasia of internal carotid arteries. Stroke in a young adult complicated by oral contraceptives.. Arch Neurol.

[13] Horne TW (1975). Fibromuscular dysplasia of the iliac arteries.. Aust NZ J Surg.

[14] Labropoulos N, Manalo D, Patel N, Tiongson J, Pryor L, Giannoukas A (2003). Uncommon leg ulcer in the lower extremity.. J Vasc Surg.

